# Estimation of heart dose in left breast cancer radiotherapy: Assessment of vDIBH feasibility using the supervised machine learning algorithm

**DOI:** 10.1002/acm2.14595

**Published:** 2024-12-06

**Authors:** Shriram Ashok Rajurkar, Teerthraj Verma, Rajeev Gupta

**Affiliations:** ^1^ DHR‐ICMR JRF, Department of Radiation Oncology KGMU Lucknow Uttar Pradesh India; ^2^ Department of Radiation Oncology KGMU Lucknow Uttar Pradesh India

**Keywords:** Breast cancer, EBRT, Heart dose, Machine learning in EBRT, Maximum heart distance, Radiotherapy, vDIBH

## Abstract

**Background and objective:**

The volunteer deep inspiration breath hold (vDIBH) technique is used to reduce the heart dose in left breast cancer radiotherapy. Many times, it is faced that despite rigorous exercise and training, not all patients get benefited as expected. The primary objective of this study was to develop a machine learning program for prediction of mean heart dose before left breast radiotherapy under vDIBH.

**Methods:**

The present work is based on the dosimetric parameters of eighty‐two left breast cancer patients, who have undergone modified radical mastectomy, enrolled for their radiation treatment. The trained machine learning algorithm employed linear regression to establish a correlation between Haller Index and heart mean dose (HMD) received during the ca left breast cancer radiotherapy. Subsequently, HMD values were used to model the regression relationship with maximum heart distance (MHD).

**Results:**

The method adopted is beneficial in patient selection and assessment for suitability of patients’ radiotherapy planning under vDIBH treatment technique. For data from 21 test patients, the mean of HMD obtained from the treatment planning system (TPS) and the mean of predicted HMD by developed program were found to be 468.76 cGy and 464.66 cGy, respectively.

**Conclusion:**

The present work facilitates precise HMD prediction in left breast cancer radiation therapy even before starting the treatment planning process. Additionally, this program offers suggestions in terms of modifications in treatment settings for even better results of vDIBH techniques if not matches with the anticipated results.

## 1 INTRODUCTION

In recent decades, breast cancer has emerged as one of the most common fatal diseases worldwide in females. Its management includes various treatment methods, such as chemotherapy, surgery, radiotherapy, and immunotherapy, often used in combination with two or more. In radiotherapy, technological advances have expanded, in both treatment planning and treatment delivery modalities. Radiation therapy (RT) has undergone several technological advances, including image‐guided radiation therapy (IGRT), volumetric‐modulated arc therapy (VMAT), and intensity‐modulated radiation therapy (IMRT).^1^ Notwithstanding, several epidemiological studies have shown that many of the complications such as cardiovascular mortality and morbidity in breast cancer radiotherapy are long‐term complications in the case of left breast cancer radiotherapy.^2^, ^3^ Radiation‐induced cardiomyopathy (RICM) has been recorded at a 40‐year cumulative incidence rate of 24.8%, although the vast majority of these cases arise from cardiovascular diseases such as valvular disease or myocardial infarction (MI).^4^ The incidence of RICM increases beyond 5 years, with the potential for development even several decades after initial RT.^5^ Despite these unexpected complications, radiotherapy and, especially, three‐dimensional conformal radiation therapy (3DCRT) is still clinically popular as compared to the IMRT treatment technique, and is considered a superior treatment modality for breast cancer management.^6^ In breast cancer radiotherapy, the target dose is usually found to be between 40 Gy to 50 Gy, in which part of the heart may be exposed to radiation, resulting in cardiac toxicity. There are several risk factors associated with radiation‐induced cardiovascular disease. During breast cancer radiotherapy, the radiation dose to the mediastinum can damage any component of the heart such as myocardium, coronary, pericardium, valves, and conduction system resulting in cardiovascular complications.^7^ The Stockholm research team reported that, compared to breast cancer patients who underwent surgery alone, those who received postoperative radiotherapy showed a significant increase in mortality due to ischemic heart disease.^8^ The study by Darby et al. showed that left‐sided breast cancer patients have a higher risk of radiation‐related cardiotoxicity than right‐sided breast cancer patients.^9^

Many of the advanced treatment strategies, such as partial breast irradiation^10^ and other sophisticated procedures such as breath hold techniques,^11^, ^12^ have been adopted to reduce cardiovascular toxicity. One of the popular and non‐invasive technique is volunteer deep inspiration breath hold (vDIBH) technique. The implementation of vDIBH requires additional time and dedicated staff compared to the conventional treatment approach, which makes it difficult due to the high workload in the radiotherapy department, especially with the low featured facilities. This ultimately results in an increase in patient waiting time and the patient queue also increases.^13^ Many times, it is faced that despite giving rigorous exercise and training to the patients, not all patients get the same benefit from it.^14^ The above‐mentioned issues have motivated the authors to attempt to develop AI program modules to address vDIBH‐associated issues faced during its implementation. The primary objective of this study was to develop an innovative AI tool capable of predicting mean heart radiation dose before actual radiotherapy treatment planning begins. Additionally, this tool offers recommendations regarding the suitability of employing vDIBH technique in left breast cancer radiotherapy, based on anatomical measurements derived from diagnostic/therapeutic computed tomography (CT) scans taken under free breathing setup.

## 2 MATERIALS AND METHODS

### 2.1 CT simulation and treatment planning

#### 2.1.1 Free breathing setup

**FIGURE 1:**
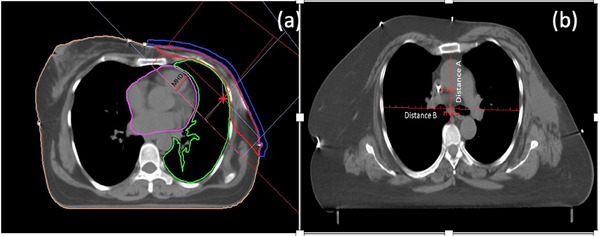
Tangential field & maximum heart distance (a), and transverse slice showing distance A & B used in the Haller Index calculation (b).

The present study is a retrospective work based on the dosimetric parameters of eighty two left breast cancer patients, who have undergone modified radical mastectomy, treated in the department of radiotherapy, enrolled for their radiation treatment during March 2022 to September 2023. The patients enrolled in this study underwent three‐dimensional conformal radiotherapy (3DCRT), with either forward planed fixed gantry with field in field technique or wedged plan as per the requirement of the discrete patient anatomy. All the patients in free breathing underwent CT scan imaging having transverse slice thickness of 5 mm for the purpose of radiotherapy planning using a CT simulator (Brilliance; Philips, Netherland). The radiotherapy treatment planning was done employing 6 MV photon beams produced by the linear accelerator (Synergy; Elekta Medical System, Sweden) using the treatment planning system (Xio, Elekta Medical System, Sweden). Dose calculation was carried out using superposition algorithm. The prescribed dose for all patients was 42.56 Gy delivered over 16 fractions, five fractions per week and 2.66 Gy/day over five consecutive days per week keeping source to axis distance (SAD) technique. All the patients were planned and treated using the two tangentially opposed fields, in addition to one anterior supraclavicular field as shown in Figure 1a. The orientation of two tangential fields was finalized keeping the aim that minimum heart and lung volume would be within the radiation fields, while planning treatment volume (PTV) must have received at least 95% of the prescribed dose. Several important dosimetric parameters such as HMD, central lung distance (CLD), bridge separation (BS), maximum heart distance (MHD) in tangential fields were obtained from the treatment plans.

#### 2.1.2 vDIBH setup

A patient was enrolled for RT under vDIBH as part of her breast cancer treatment (after MRM), with the aim of validating the recommendation from the developed algorithm. This was done after obtaining necessary ethical approval from the institutional ethics cell vide letter no. 113th ECM IIA/P17. In this case, the CT scan imaging of the region of interest of the patient was acquired, that is, simulated, both in the deep inhalation breath hold and free breathing setups. The remaining processes were carried out in the same way as described in the free breathing setup for radiation delivery.

### 2.2 Machine learning database

The present study is an attempt to predict the mean dose to the heart based on anatomical dimensions obtained from a CT image set using a linear regression machine learning (ML) algorithm. ML, a subset of artificial intelligence, is a branch of computational science that analyzes, interprets, and improves a system and helps in making decisions with little or no human intervention. The ML algorithm is classified into three types, namely, supervised, unsupervised, and reinforcement.^15^ Linear regression belongs to the category of supervised ML algorithms. Supervised learning helps solve real‐world computational problems by guiding the system or data as an mentor. It involves predetermining accurate data paired with their corresponding accurate outputs, which are stored as training data sets to generate accurate outputs.^16^

### 2.3 Linear regression

Linear regression (LR) analysis is a predictive modeling technique to determine the relationship between two variables.^17^ LR is a statistical model, which establishes a relationship between dependent variable and independent variable. If the slop of a given regression line steeply increases linearly, then it is called as a positive regression line. If the regression line tends to have steeply decreasing slop, then it is said to be a negative regression line. In the present study, the ML model was implemented using the Python library's Scikit‐Learn, Pandas, NumPy, and Matplotlib. The model was developed by utilizing the dosimetric data including heart mean dose (HMD) and MHD, etc., recorded from treatment plan. The Scikit‐learn library was used to deal with dosimetric data and perform a polynomial feature function to generate a novel AI tool to predict the mean dose to the heart.

### 2.4 Relation between anatomic dimensions and MHD

The authors applied the concept of the Haller Index (HI) to investigate the relationship between anatomical dimensions and the HMD. The transverse diameter of the chest and the shortest distance between the sternum and the vertebra are the two parameters that make up the HI index.^18^ The ratio of the lateral diameter of the chest within the rib cage (“A”) to the distance between the sternum and vertebral body (“B”) at the point of maximal depression, as illustrated in Figure 1b, was used to calculate the HI.

(1) $$\mathrm{Haller}\;\mathrm{Index}=\frac{A}{B}$$

Distance “A” was evaluated as the measurement from the innermost aspect of the rib or intercostal muscle to the corresponding point on the mid‐axillary line on that specific slice. Distance “B” was derived by the measurement from the back side of the sternum on that slice to the foremost point on the vertebral body. Stahl et al. reported that HI is proportional to mean heart dose and vice versa.^19^ The authors attempted to develop an AI tool to predict the average dose to the heart by taking into account the relationship between the HI and the mean dose to the heart. The algorithm was trained using predefined dosimetric data and corresponding HI values for individual patients. Additionally, authors could successfully established a correlation between the HMD and the MHD as well. As depicted in Figure 1b, MHD is defined as the distance from the anterior cardiac contour to the posterior edge of the tangential field.^20^

### 2.5 Input data for the developed model

**FIGURE 2:**
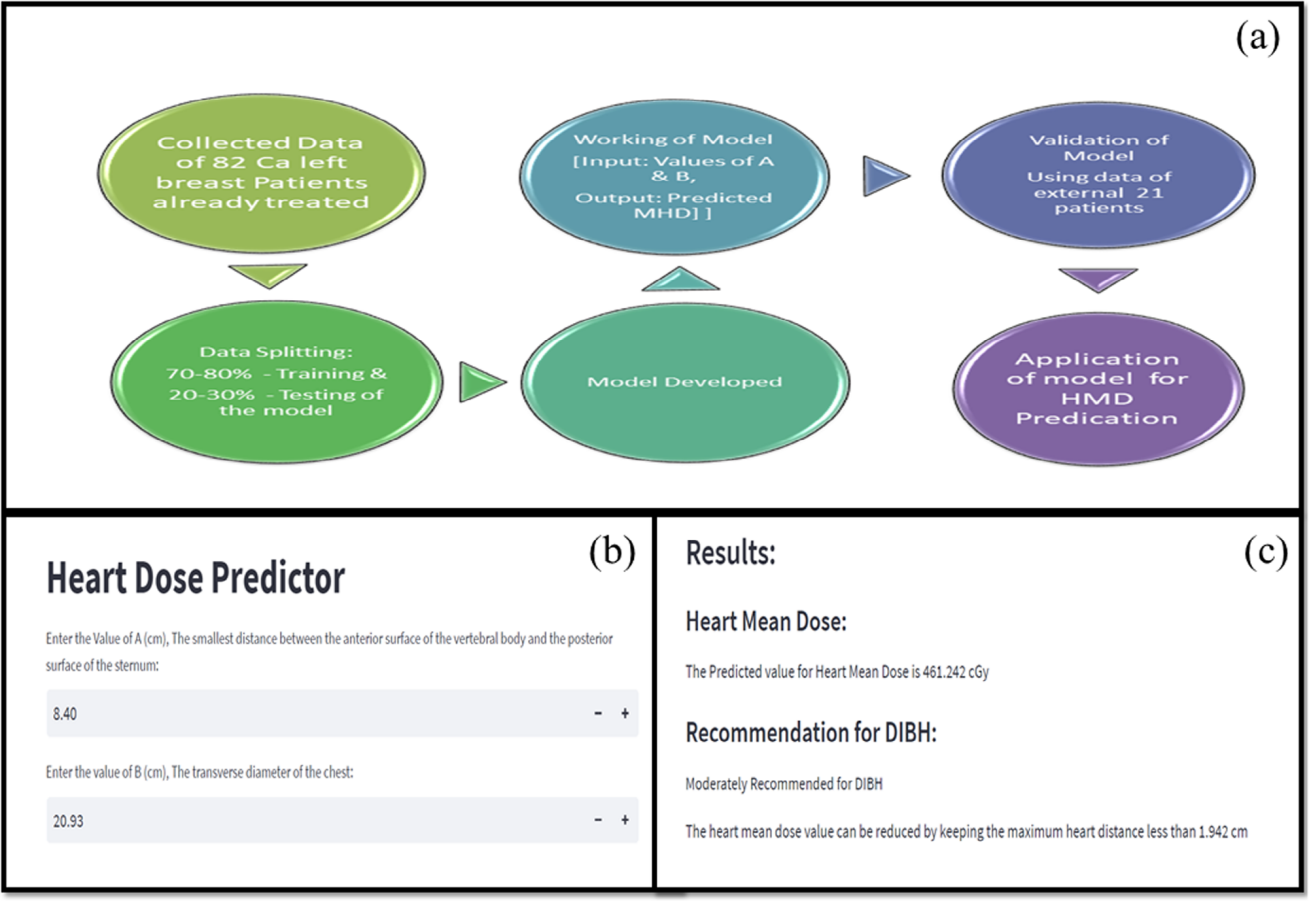
Work flow of development and validation of the HMD prediction model (a), programme input data interface (b), output interface of the programme (c).

The model in the present study was developed in such a way that user need to have only values of “A” and “B” as the input data. The work flow of development and validation of the HMD prediction model is given in Figure 2a. This developed AI tool itself calculates the HI using the input data. The developed supervised ML algorithm employs linear regression to establish a correlation between HI and the mean dose received by the heart during the ca left breast cancer radiotherapy. Subsequently, HMD values were used to model the regression relationship with MHD.

## 3 RESULT

The developed model was validated and evaluated for the purpose of prediction of HMD in case of left breast cancer radiotherapy planning using two parallel opposite tangential fields as shown in Figure 1b.

As soon as program is provided the input, that is, values of parameters A & B as shown in Figure 2b, the program developed in the present study, after successive iterations, provides the output, as shown in Figure 2c. In addition to HMD dose prediction, the developed program facilitates recommendations about the suitability of the individual patient for its treatment using the vDIBH techniques in connection to optimum benefit to the patient of vDIBH technique in terms of reduction in the dose to heart and lung even prior to the actual radiotherapy planning and subsequently during radiation delivery.

In the present study, treatment planning parameter of twenty one left breast cancer patient was taken to evaluate the performance of the developed program and compared its predicted value with the corresponding HMD values recorded from the treatment plan, which was used for the radiotherapy of real patients as shown in Table 1.

**TABLE 1:** Heart mean dose recorded from the treatment planning system and by developed model for the corresponding A & B values.

Sample No.	Distance A	Distance B	Algorithm calculated HI values	Heart mean dose value from treatment plan (cGy)	Predicted heart mean dose values (cGy)
1	8.25	21.10	2.557	514	465
2	7.65	21.66	2.831	473	483
3	10.7	21.30	1.990	313	430
4	7.25	20.75	2.862	551	486
5	7.52	21.16	2.813	537	482
6	7.89	20.38	2.583016	468	467
7	8.90	20.43	2.295506	428	449
8	9.20	22.19	2.411957	405	456
9	8.15	21.64	2.655215	498	472
10	8.01	19.27	2.405743	457	456
11	9.25	22.68	2.451892	411	459
12	6.55	20.68	3.157252	574	503
13	9.32	18.89	2.026824	577	432
14	9.28	18.94	2.040948	502	433
15	7.86	19.61	2.494911	512	461
16	7.32	19.58	2.674863	518	473
17	7.12	21.57	3.029494	545	495
18	8.35	22.36	2.677844	413	473
19	6.90	21.58	3.127536	558	501
20	10.12	18.67	1.844862	184	420
21	8.40	20.93	2.491666	406	461

### 3.1 Statistical analysis

Statistical analysis of all the data under the present study was performed using the Python program (version 3.9.12) developed on Visual Studio Code (version 1.84.2). The developed Python code for statistical analysis and hypothesis testing is available on GitHub; the respective link is provided in the source code availability section of the this manuscript.

### 3.2 Performance analysis of in‐house developed heart dose prediction model

**FIGURE 3:**
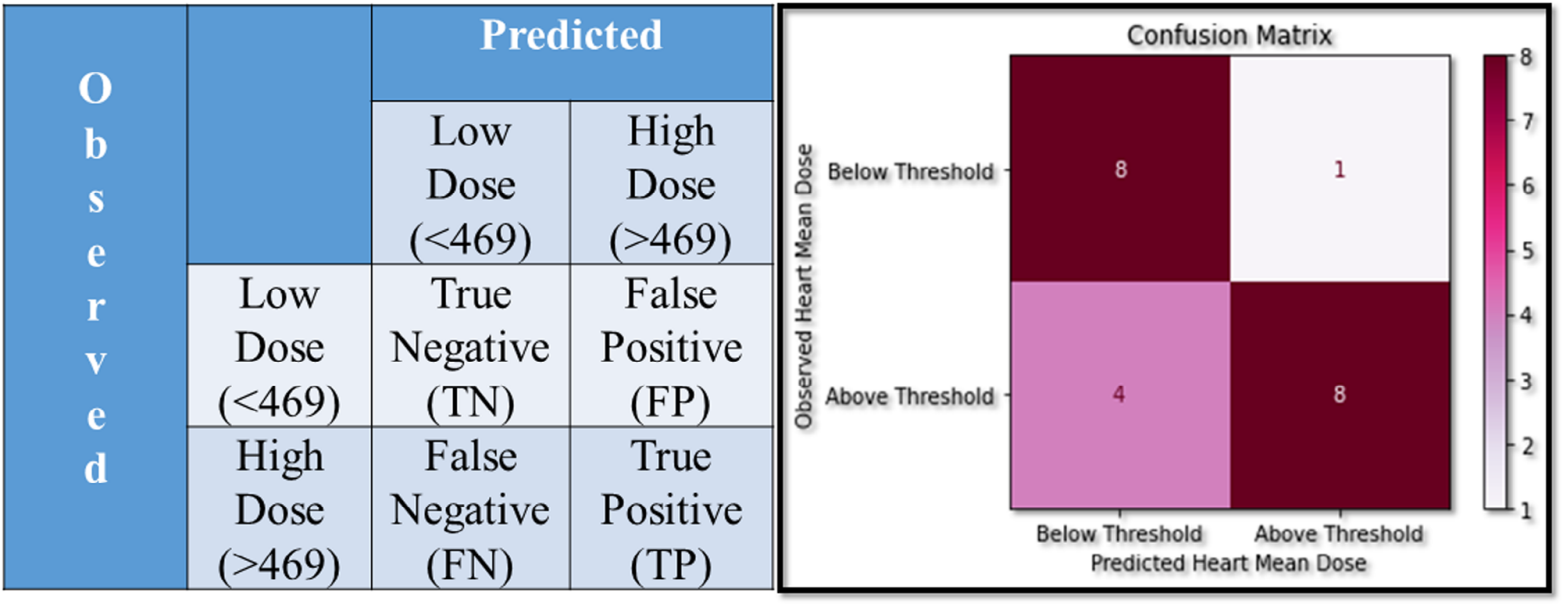
Statistical analysis program generated confusion matrix.

The performance analysis of the developed model was evaluated by using the confusion matrix as shown in Figure 3 in terms of the true positive (TP), false positive (FP), false negative (FN), and true negative (TN). First threshold value was determined by using observed HMD values from observation Table 1. The threshold value is calculated by using Equation (2).

(2) $$\begin{array}{cc} & \mathrm{Threshold}\;\left(\mathrm{Mean}\;\mathrm{Observed}\;\mathrm{Value}\right)\\ & \;=\frac{\left(\mathrm{Sum}\;\mathrm{of}\;\mathrm{Observed}\;\mathrm{Values}\right)}{\left(\mathrm{Number}\;\mathrm{of}\;\mathrm{Samples}\right)} \end{array}$$

Based on the output of Equation (2), the parameters, namely, TP, FP, FN, and FP are defined as follows.

1. True positive (TP): where both observed and predicted values are above the threshold.
2. False positive (FP): where the predicted value is above the threshold but the observed value is below the threshold.
3. True negative (TN): where both observed and predicted values are below the threshold.
4. False negative (FN): where the predicted value is below the threshold but the observed value is above.

Statistical parameter,^21^ as given in Equations ((3), (4), (5), (6)), for the performance assessment of the developed HMD prediction model are given as follows.

(3) $$\mathrm{Precision}=\frac{TP}{TP+FP}$$

(4) $$\mathrm{Recall}=\frac{TP}{TP+FN}$$

(5) $$\mathrm{Specificity}=\frac{TN}{TN+FP}$$

(6) $$\mathrm{Accuracy}=\frac{TP+TN}{TP+TN+FN+FP}$$

Keeping the threshold value 468.761 cGy, the result of the HMD predictive model was recorded to be of accuracy 0.76, with the precision rate of 0.88, a recall rate of 0.66, and a specificity rate of 0.66.

The statistical analysis program generated the confusion matrix as shown in Figure 3.

### 3.3 Hypothesis testing using Python programming

Initially, the null hypothesis was set to state that there is no considerable difference between the observed and predicted values of the HMD as shown in Table 1, considering the *p*‐value less than 0.05, statistically significant. The paired *t*‐test was used to measure the difference between the observed and predicted values and same is expressed in terms of standard errors.

The output of the developed program for above‐mentioned hypothesis testing includes the mean of the observed value, the mean of the predicted value, the absolute differences between the mean of the observed and predicted HMD, *p* values and the paired *t*‐test value. In this case, the *t*‐statistic is approximately 0.230, which suggests that the observed and predicted values are about 0.230 standard errors apart. In the present study, the *p*‐value was approximately 0.819, which iterates that the probability of obtaining a *t*‐statistic as extreme as 0.230. Since the *p*‐value is greater than the chosen significance level, so authors do not have enough evidence to reject the null hypothesis, assuming the null hypothesis is true. This suggests that, based on the paired *t*‐test, there is not sufficient statistical evidence to conclude that the observed and predicted values are significantly different from each other.

The mean of observed HMD and the mean of predicted HMD were found to be 468.76 cGy and 464.66 cGy, respectively, having the absolute difference of 4 cGy out of 21 patients.

## 4 DISCUSSION

The goal of radiotherapy is to deliver precise and adequate dose to the tumor avoiding excess dose to surrounding normal tissue.^22^ The effects of radiation on the heart are a potentially noteworthy and serious clinical concern in breast cancer radiotherapy treatment.^23^ As such, no precise threshold relationship is found between mean heart dose and the risk of corresponding coronary events.^20^ However, according to Darby et al., there is a 7.4% lifetime risk increase of coronary events for every 1 Gy mean radiation dose escalation to the heart.^9^ The field of radiation applications has witnessed a remarkable surge in the utilisation of artificial intelligence and ML applications recently.^24^

There are numerous studies which have attempted to develop prediction model of HMD in breast cancer radiotherapy.^25^, ^26^, ^27^ Out of four methods—decision trees, random forests (RF), XGB, and deep neural networks (DNNs)—based on information from a single CT image slice and clinical variables, Kamizaki et al. observed that the DNN was more accurate in predicting the HMD without taking the HI into account.^28^

However, in the current study, the authors developed a user‐friendly regression model that just uses two parameters—A and B to predict mean heart dose.

Considering of the time‐consuming nature of radiation planning and execution for the vDIBH, skilled personnel and dedicated time for this exercise are required. Thus, by simply recording the values of A and B parameters, the approach suggested in this study for assessing the suitability of the vDIBH technique prior to beginning of the treatment planning itself would be very beneficial to improve the throughput of the radiotherapy facility.

It is to the best of the author's knowledge, the first comprehensive software program of its kind ever developed, which is freely accessible and very user‐friendly and does not require any deep knowledge of programming to use it. This heart dose prediction program can be installed in the TPS itself or in the other computer side by side.

Initially, the external data set of 21 patients was used in this study to validate the proposed program. Following that, one case of left breast cancer (sample no. 21) was selected for the purpose of study.

The measurements for parameters A and B, which in this case measured 8.40  and 20.93 cm, respectively, were determined using the CT data. These values were the input data for the heart dose prediction program, as shown in Figure 2.

Through successive iterations, this model generated the following output:

“The predicted value for heart mean dose is 461.242 cGy, which is moderately recommended for DIBH. The heart mean dose value can be reduce by keeping the maximum heart distance less than 1.942 cm.”

**FIGURE 4:**
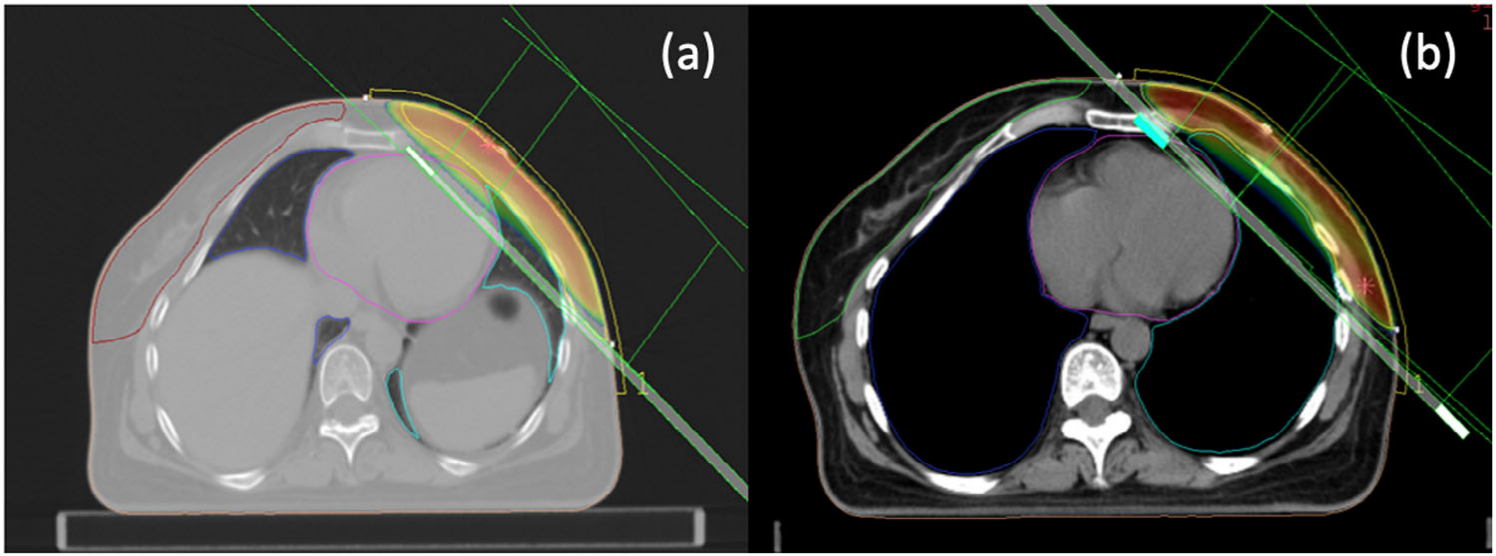
(a) Involvement of heart partial volume under radiation beam arrangement in free breathing CT (a): and vin DIBH CT.

To evaluate the recommendation of DIBH program, current case study scheduled for vDIBH treatment after taking informed consent form from the patient. Two treatment plans were prepared: free breathing and DIBH treatment plans, respectively, as shown in Figure 4. In a comparison of dosimetric data of both treatment plans, the observed HMD was 406 cGy in the FB plan. The difference between predicted and observed HMD was found to be 55 cGy. The vDIBH treatment plan was formulated for the same case, ensuring that the MHD remained below 1.95 cm. The same patient (in the example) under the DIBH plan produced a mean heart dose of just 209 cGy with a MHD of 1.42 cm. This demonstrates that, in comparison to the FB treatment plan, the HMD in the vDIBH plan was reduced by about 51.5%.

Since the HI is measured manually by the program user, its value may vary in the sense of interpersonal measurements variability due to one or more factors, such as the selection of a slice for parameters A and B measurements without following the correct criteria for the same, differences in the measurement of parameters A and/or B, etc. This cannot be denied to happen in a radiotherapy facility specially to one heavy patient loaded department having fresher radiation professionals, such as medical physicists, radiation oncologists, and dosimetrists. Keeping this fact into consideration, the sensitivity of the HI measurement to its effect on the prediction accuracy of the HMD was investigated. For this, the same CT data set of a Ca left breast cancer patient was given to nine users separately to measure parameters A and B according to the criteria given under section 2.4 of this article. Analysis of recorded data by nine users showed that the variability in HI values came with a standard deviation of 0.070 with the prediction results of the HMD having a standard deviation of only 4.38 cGy as shown in Table 2. This shows that the predicted outcome is not that much sensitive to interpersonal variability in A & B parameter measurements among professionals. This was done keeping in mind the very practical issue of interpersonal skill levels among the users in the same department toward using the program.

**TABLE 2:** Observations of the users for HI sensitivity to HMD prediction.

Program users	A (cm)	B (cm)	HI	Predicted MHD (cGy)
User 01	6.71	20.15	3.00	493.5
User 02	6.77	20.48	3.02	494.9
User 03	6.58	20.14	3.06	497.1
User 04	7.21	20.64	2.86	484.6
User 05	7.13	20.54	2.88	485.7
User 06	7.12	20.51	2.88	485.7
User 07	6.93	20.48	2.95	490.4
User 08	6.86	20.38	2.97	491.4
User 09	6.91	20.46	2.96	490.8
Standard deviation			0.06915	4.3832

### 4.1 Optimization of correlation

Nonetheless, the program designed to calculate HI based on, general human, representative anatomical dimensions was used by the authors of the current exploratory study as input data. In the present study, regression models were employed to predict the mean dose to the heart and offers recommendations for suitability of vDIBH based on anatomical dimensions of the patient under study. This model was developed using regression algorithm in which 70%–80% data served as training data and 20%–30% as testing data.

**FIGURE 5:**
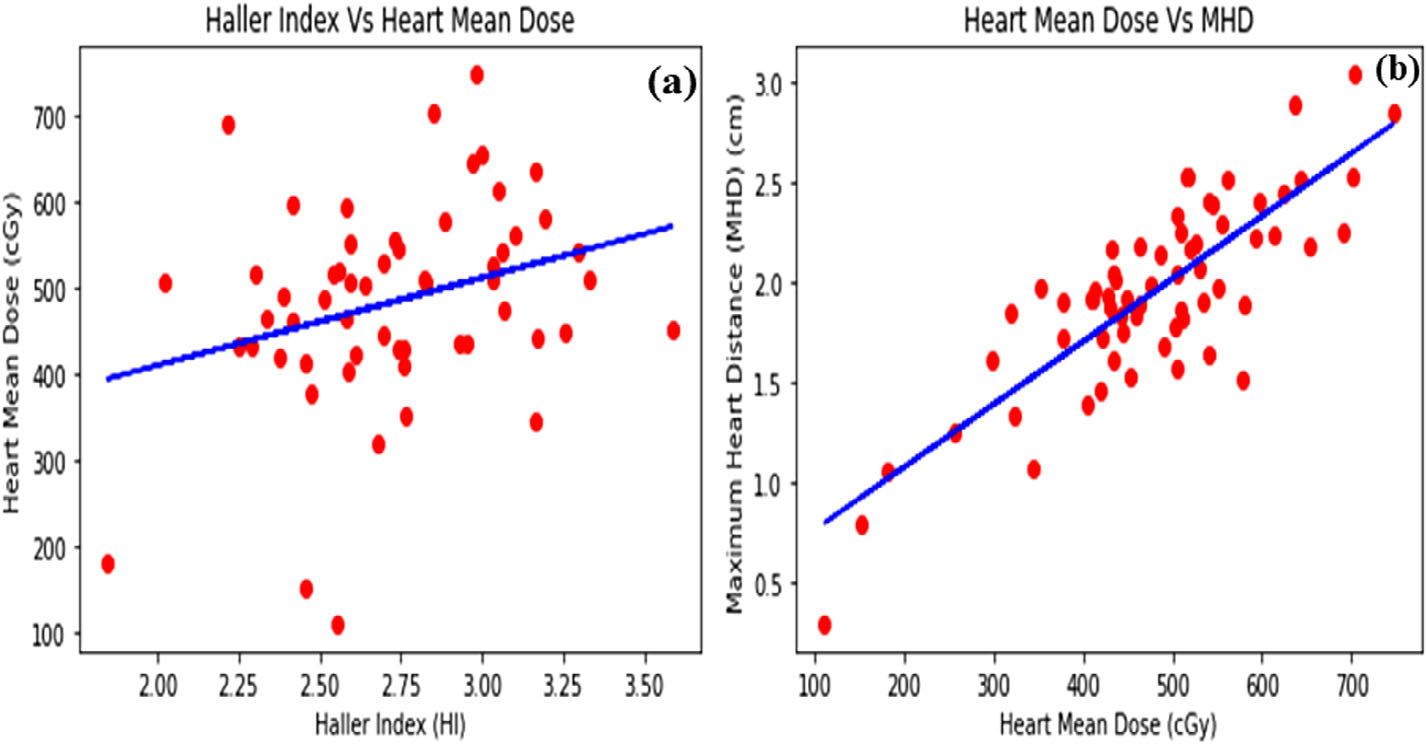
The linear regression distribution between HI & heart mean dose (a), and heart mean dose and maximum heart distance (b).

The training and testing data were kept in the ratio of 80:20 for the relationship between HMD and MHD while 70:30 for the relationship between HI and HMD. The *r* value of 0.294 was found to be consistent with the correlation between HI and mean dose to heart under the regression model with a positive correlation between the two quantities as shown in Figure 5a.

Similarly, as obvious from Figure 5a, with the increase in HI, the HMD tends to increase. Also, the same trend of variation, having an *r* value 0.820, was observed for the regression relation between HMD and MHD, as shown in Figure 5b. The regression correlation highlights a positive relation between HMD and MHD, indicating that as the MHD increases, the HMD also tends to increase.

### 4.2 Limitations

Breast volume has a major impact on HMD, while patients of the Asian ethnicities typically have lower breast volumes than patients from the US and Europe.^29^, ^30^ Therefore, the HMD tends to be higher in the United States and Europe patients as compared with the Asian ethnicity patient.^29^, ^31^, ^32^ The present model was developed by taking the Indian patient data that belongs to Asian countries, which means the model will not be as useful for patients who belong to the US and Europe. In order to improve this program, data from various regions of other countries must be collected to facilitate more precise output result for the intended patients. It is required to collect data from different regions of other countries so it will provide a more accurate output result for desired patients.

## 5 CONCLUSION

The present study facilitates precise prediction of the HMD, using the anatomical dimensions obtained from the patient's therapeutic or diagnostic CT imaging, before the actual process of radiation treatment planning in left breast cancer radiotherapy begins.

Following program recommendations, this approach could potentially be beneficial in the process of patient selection and suitability for radiation planning under the vDIBH treatment regimen. Furthermore, the program may help the oncology team to minimize unnecessary efforts and save time in the matter of implementation of vDIBH treatment.

## 6 SOURCE CODE AVAILABILITY

This AI tool is available on GitHub. The same can be assessed using link https://github.com/RGBRAND/Heart‐Dose‐Prediction‐Model. This program is open source, that is, accessible to all radiation oncology professionals and is free for its intended use subject to citation of this article when finding are published.

The developed model is compatible with all operating systems. Follow these steps to execute the program:

1. Set up a Python environment on your system.
2. Open the provided file in the Python environment.
3. To run the program, start by opening the terminal and using the command “clt+J.”
4. Type the following command: “streamlit run Heart_dose_predcitor.py” and press enter.
5. After entering the above command, the program will run automatically, launching in your system's default web browser.
6. Subsequently, the program interface will be displayed in the form of a user‐friendly app.

Note: Ensure that you have the necessary dependencies installed and configured in your Python environment before executing the program.

## AUTHOR CONTRIBUTIONS

Shriram Ashok Rajurkar, Program development and data collection, initial drafting; Teerthraj Verma—Conceptualizing the idea, designing the objectives and method of execution of the idea, programming and draft finalization of the presented work. Rajeev Gupta—Clinical input, Review of draft.

## CONFLICT OF INTEREST STATEMENT

The authors declare no conflicts of interest.
